# Effect of Additional Surface Conditioning Methods and Adhesive Systems on Microleakage at Composite Repair Interfaces: An in Vitro Study

**DOI:** 10.3390/ma19194149

**Published:** 2026-09-29

**Authors:** Iryna Ushatkina, Zuzanna Grzech-Leśniak, Marek Milcarz, Katarzyna Skośkiewicz-Malinowska, Jacek Matys, Kinga Grzech-Leśniak

**Affiliations:** 1 EMDOLA, Wroclaw Medical University, Krakowska 26, 50-425 Wroclaw, Poland; ushatkina@gmail.com (I.U.); milcarzdom@interia.pl (M.M.); 2 Department of Experimental Dentistry, Faculty of Medicine and Dentistry, Wroclaw Medical University, Krakowska 26, 50-425, Wroclaw, Poland; zuzanna.grzech-lesniak@student.umw.edu.pl; 3 Department of Conservative Dentistry and Endodontics, Faculty of Medicine and Dentistry, Wroclaw Medical University, 50-425 Wroclaw, Poland; katarzyna.skoskiewicz-malinowska@umw.edu.pl; 4 Dental Surgery Department, Faculty of Medicine and Dentistry, Wroclaw Medical University, Krakowska 26, 50-425 Wroclaw, Poland; 5 Department of Integrated Dentistry, Faculty of Medicine and Dentistry, Wroclaw Medical University, Krakowska 26, 50-425 Wroclaw, Poland; kinga.grzech-lesniak@umw.edu.pl; 6 Department of Periodontics, School of Dentistry, Virginia Commonwealth University, Richmond, VA 23298-0566, USA

**Keywords:** composite repair, microleakage, Er:YAG laser, airborne-particle abrasion, universal adhesive, resin composite, in vitro

## Abstract

Repair of aged resin composites requires additional surface conditioning and an adhesive protocol capable of limiting interfacial leakage. This in vitro study evaluated three additional conditioning methods and two adhesive systems at aged composite–new composite repair interfaces. Ninety extracted posterior teeth were restored, thermocycled, and partially prepared with a diamond bur to create standardized repair surfaces. The surfaces then received additional diamond-bur roughening, 50-µm Al_2_O_3_ airborne-particle abrasion, or Er:YAG laser irradiation and were treated with Clearfil SE Bond or Clearfil Universal Bond Quick 2 (n = 15 per combination). After repair and additional thermocycling, maximum linear dye penetration was measured as the primary outcome; a derived ordinal grade was analyzed as a supportive, exploratory secondary outcome. A two-factor permutation analysis detected no significant effect of conditioning method (*p* = 0.142), adhesive system (*p* = 0.606), or their interaction (*p* = 0.341) on linear microleakage. In the ordinal proportional-odds analysis, the method effect was not statistically significant (*p* = 0.053); neither the adhesive effect (*p* = 0.763) nor the interaction (*p* = 0.754) was significant. Under the tested conditions, statistically significant differences in microleakage were not detected among the evaluated combinations.

## 1. Introduction

Resin-based composites are among the most frequently used restorative materials in contemporary conservative dentistry. Their clinical use is supported by favorable aesthetics, adhesive application, and the possibility of preserving dental hard tissues. Nevertheless, composite restorations are exposed to thermal changes, occlusal loading, water sorption, pH fluctuation, enzymatic degradation, and wear. These processes may contribute to marginal deterioration, discoloration, chipping, fracture, and loss of interfacial integrity [1]. Recent studies likewise show that water sorption and solubility, composite formulation, and curing conditions can modify the properties of resin-based materials [2,3,4].

When a composite restoration is partially defective, the clinician may either replace the entire restoration or repair the defective area. Complete replacement can remove sound tooth structure, enlarge the cavity, and weaken the remaining tooth. In deep restorations, it may also increase the risk of pulpal injury. Repair is therefore consistent with Minimal Intervention Dentistry when the remaining restoration is clinically acceptable [5,6]. Successful repair requires durable adhesion between an aged composite substrate and the newly placed material. Fresh composite layers can interact through the oxygen-inhibited layer and residual unreacted methacrylate groups. By contrast, an aged surface contains fewer available reactive groups and may exhibit water sorption, contamination, and degradation of the resin matrix. Mechanical and chemical pretreatment is consequently used to improve wetting, micromechanical retention, and interfacial bonding [7,8]. Composite age, substrate condition, and material formulation can further influence bonding behavior [9,10,11].

Microleakage is the penetration of fluids, ions, molecules, or bacteria through microscopic interfacial pathways. At a composite–composite repair interface, leakage may contribute to marginal staining and deterioration of the repaired region. Dye penetration remains a common comparative laboratory method, although its two-dimensional nature, dependence on sectioning, and limited relationship with clinical longevity restrict interpretation [12,13,14]. Confocal and dye-based studies further demonstrate that measured leakage depends on the restorative material and experimental protocol [15,16]. Mechanical preparation methods include diamond-bur roughening and airborne-particle abrasion. A diamond bur creates macroscopic grooves but may leave an irregular smear layer. Airborne-particle abrasion with aluminum oxide produces a more diffuse microretentive topography and exposes filler particles. Reported repair outcomes vary according to composite composition, aging, particle size, pressure, treatment duration, and the subsequent adhesive protocol [8,17]. Studies of aged composite repairs have also reported protocol-dependent effects of air abrasion, bur roughening, laser treatment, and composite type [18,19,20]. Universal adhesives are attractive because they simplify repair procedures and may contain functional monomers such as 10-methacryloyloxydecyl dihydrogen phosphate. Some formulations also contain a silane coupling agent. Evidence regarding the benefit of silane incorporated within a universal adhesive remains inconsistent and appears to depend on filler exposure, substrate composition, adhesive acidity, storage conditions, and whether a separate silane is used [21,22,23]. Repair-specific studies indicate that universal-adhesive composition, silanization, mechanical pretreatment, and aging can alter bond strength and fatigue behavior [24,25,26,27]. Other adhesive studies further show that application technique and substrate pretreatment can modify bond strength [28,29]. Importantly, comparison of two different adhesive systems cannot isolate the effect of silane because the products also differ in monomers, solvents, filler content, and application procedure.

Er:YAG laser irradiation has been proposed as a non-contact method of modifying aged composite surfaces. Its effect is strongly dependent on pulse energy, repetition rate, pulse duration, water spray, focal distance, spot size, scanning speed, and the optical and thermal properties of the composite. Published studies have reported both favorable and unfavorable repair outcomes; laser conditioning therefore cannot be assumed to be superior to conventional methods without protocol-specific evidence [30,31,32,33,34]. Evidence from other dental substrates and experimental models likewise demonstrates that laser effects depend on wavelength, delivered energy, exposure parameters, and the irradiated material or tissue [35,36,37,38]. Most composite-repair studies have focused on bond strength rather than leakage at the aged composite–new composite interface. Direct comparisons of diamond-bur roughening, Al_2_O_3_ airborne-particle abrasion, and Er:YAG laser irradiation within the same factorial adhesive design remain limited.

Most composite-repair studies have focused on bond strength rather than dye penetration at the aged composite–new composite interface, and direct evaluations of microleakage within a controlled 3 × 2 factorial design remain limited. The aim of this in vitro study was therefore to assess the effects of an additional surface-conditioning method, adhesive system, and their interaction after standardized diamond-bur creation of the repair surface. Maximum linear dye penetration was designated as the primary outcome, and the ordinal leakage grade derived from the same measurement was considered a supportive, exploratory secondary analysis rather than an independent endpoint. The null hypotheses were that the additional conditioning method, adhesive system, and their interaction would not significantly affect microleakage.

## 2. Materials and Methods

### 2.1. Study Design, Sample Selection, and Ethical Considerations

The study was approved by the Bioethics Committee of the Wroclaw Medical University, approval number KB 318/2025, dated 25 November 2025.

Ninety (n = 90) extracted human posterior teeth, including 12 premolars and 78 molars, were used in the study. The teeth had been extracted for reasons unrelated to the present study, such as orthodontic, periodontal, prosthetic or surgical indications. All biological material was anonymized before use.

#### 2.1.1. Inclusion Criteria

- Intact clinical crowns;
- Absence of previous restorations;
- Absence of visible carious lesions;
- Absence of visible cracks or fractures;
- Sufficient crown structure to allow for standardized Class II cavity preparation;
- Storage conditions compatible with the study protocol.

#### 2.1.2. Exclusion Criteria

- Visible carious lesions;
- Previous restorations;
- Enamel cracks;
- Crown fractures;
- Extensive pathological wear;
- Hypoplasia, erosion or other surface alterations that could interfere with cavity preparation.

### 2.2. Sample Size Calculation

The sample size was estimated a priori for the primary outcome of maximum linear dye penetration using G*Power version 3.1.9.4 (Heinrich Heine University Düsseldorf, Germany). The calculation used the F-test family and a fixed-effects omnibus one-way ANOVA with six groups, an a priori large effect size of Cohen’s f = 0.40, α = 0.05, and 80% power. Because no sufficiently comparable pilot dataset was available, the large effect size was selected a priori as the minimum difference considered detectable within the available in vitro design rather than being derived directly from the sample sizes of previous studies. The calculated minimum total sample size was 86. This was increased to 90 to obtain six balanced groups of 15 specimens and allow for potential technical exclusions. This calculation addressed a six-group omnibus effect and was not designed to provide specific power for the factorial interaction; accordingly, smaller main or interaction effects may have gone undetected.

### 2.3. Specimen Preparation

After extraction, the teeth were cleaned of soft-tissue remnants and deposits and stored in 0.2% thymol at room temperature for 1–3 months. Before preparation, specimens were rinsed, cleaned with pumice for 10 s, rinsed again and gently air-dried.

### 2.4. Materials

The principal restorative and adhesive materials are listed in Table 1.

### 2.5. Cavity Preparation and Primary Restoration

A standardized Class II cavity involving one cusp was prepared in each tooth. The buccolingual width was approximately one-third of the intercuspal distance, cavity depth was 4 mm and cusp reduction was 3 mm. Dimensions were checked with a periodontal probe.

Preparations were made with diamond burs, size 014, at 200,000 rpm, under water–air cooling at a water flow rate of approximately 30 mL/min, by one operator. A new bur was used after every five teeth.

Clearfil SE Bond was applied to the tooth substrate using the following sequence: primer application for 20 s, air-drying, bond application, air-thinning, and light curing for 10 s. CLEARFIL MAJESTY ES-2 Universal was placed in increments no thicker than 2 mm. Each increment was light-cured for 10 s with an Eighteeth Curing Pen LED unit (Changzhou Sifary Medical Technology Co., Ltd., Changzhou, China) set to a nominal irradiance of 1500 mW/cm^2^. The light guide was positioned perpendicular to and as close as practicable to the restoration surface. The unit was not calibrated with an external radiometer immediately before or during the experiment.

Restorations were finished and polished with a Kenda system.

### 2.6. Aging of Primary Restorations

After restoration, specimens were stored in distilled water for 24 h and then thermocycled for 1000 cycles between 5 ± 2 °C and 55 ± 2 °C, with a dwell time of 30 s and a transfer time of 10 s. This protocol was selected as a standardized, moderate thermal challenge consistent with laboratory microleakage and composite-repair models [17,39,40].

### 2.7. Simulation of Restoration Damage

After the first thermocycling procedure, part of the primary composite restoration was removed with a rounded tapered diamond bur with a 2-mm depth marker under water–air cooling. This standardized removal step was applied to every specimen before group allocation and created a flat aged-composite repair surface. Accordingly, the subsequent experimental procedures represented additional conditioning of an already bur-created repair surface rather than three fully independent methods of initial surface creation.

The standardized removal was performed to a depth of 2 mm, controlled by the depth marker on the bur. All operative procedures were performed by one operator.

### 2.8. Randomization and Allocation

Specimens were assigned identification numbers from 1 to 90 and allocated to six groups using one simple computer-generated randomization sequence obtained from Randomizer.org (accessed on 6 January 2026). Allocation was not stratified by tooth type. Each group contained 15 specimens, and tooth type was not used as a statistical covariate. The measured endpoint was confined to the standardized composite–composite repair interface (Table 2).

### 2.9. Surface Preparation of Aged Composite

After standardized bur removal in all specimens, the repair surfaces received the assigned additional conditioning. In Groups 1 and 2, the aged composite was additionally roughened with a coarse-grit spherical diamond bur, size 016, at 100,000 rpm for 10 s under water–air cooling. A new bur was used after every five specimens.

In Groups 3 and 4, the bur-created repair surface received additional 50-µm Al_2_O_3_ airborne-particle abrasion (Prep Start H_2_O, Danville, ZEST DENTAL SOLUTIONS, Carlsbad, CA, USA) at 4.0 bar for 10 s, using a 10-mm nozzle-to-surface distance and a 90° angle. The surface was rinsed with water–air spray and gently dried.

In Groups 5 and 6, the bur-created repair surface received additional Er:YAG laser irradiation (LightWalker, Fotona, Slovenia; wavelength 2940 nm) using an H02-N tipless handpiece in non-contact mode with water–air cooling. The handpiece outlet was maintained approximately 1 mm from the composite surface. Settings were 250 mJ per pulse, 20 Hz, 100 µs pulse duration, 5 W average power, and 10 s exposure. The nominal peak power was 2.5 kW. The reported energy density of 39.3 J/cm^2^ per pulse was a nominal value calculated from the pulse energy and the manufacturer-specified 0.9-mm focal spot; fluence at the specimen surface was not independently measured. The operator manually traversed the complete repair surface repeatedly with horizontal and vertical movements throughout the 10-s exposure. Scanning speed was not instrument-controlled, and laser output was not independently calibrated immediately before or during the experiment.

### 2.10. Composite Repair Procedure

After surface preparation, specimens received the adhesive assigned to their group. No separate silane and no phosphoric-acid cleansing step were used in any group.

In Groups 1, 3 and 5, Clearfil SE Bond primer was applied for 20 s and gently air-dried. Bond was applied uniformly, gently air-thinned and light-cured for 10 s.

In Groups 2, 4 and 6, Clearfil Universal Bond Quick 2 was rubbed over the repair surface without a waiting period, air-dried for at least 5 s until the adhesive layer no longer moved and light-cured for 10 s.

Repairs were completed with CLEARFIL MAJESTY ES-2 Universal in increments no thicker than 2 mm. Each increment was light-cured for 10 s with the LED unit set to 1500 mW/cm^2^. Repairs were finished and polished with the same Kenda system.

### 2.11. Aging of Repaired Restorations

All repaired specimens underwent a second 1000-cycle thermocycling procedure between 5 ± 2 °C and 55 ± 2 °C, with a dwell time of 30 s and a transfer time of 10 s. No mechanical loading was applied.

### 2.12. Microleakage Test

After thermocycling, the external tooth surfaces were coated with transparent nail varnish, leaving the aged composite–new composite interface exposed.

Specimens were immersed in 0.5% basic fuchsin for 24 h and then rinsed thoroughly.

Each specimen was ground in the buccolingual direction under water cooling until the geometric center of the repaired area was exposed. The same examiner selected the central plane for all specimens according to this prespecified geometric criterion. Specimens and image files were identified only by coded numbers, and the examiner was blinded to group allocation during section selection and image assessment. The prepared surface was rinsed and gently air-dried, photographed under standardized lighting with a Nikon digital camera and an AF-S Micro NIKKOR 105 mm f/2.8G lens at a fixed distance, calibrated against a reference scale, and analyzed with IC Measure software, version 4.0.0.219 (The Imaging Source Europe GmbH, Bremen, Germany) (Figure 1).

The maximum linear dye penetration along the aged composite–new composite interface was measured once in millimeters and designated as the primary outcome. No repeat measurements were performed, and intra-examiner reliability was therefore not estimated. For secondary analysis, the same linear measurement was classified using a five-grade ordinal scale adapted from Bilgrami et al. [39]: grade 0, 0.0 mm; grade 1, >0.0 to ≤0.5 mm; grade 2, >0.5 to ≤1.0 mm; grade 3, >1.0 to <2.0 mm; and grade 4, ≥2.0 mm (Table 3). Thus, the linear and ordinal variables were two representations of the same measurement and were not treated as independent confirmatory outcomes.

### 2.13. Statistical Analysis

Maximum linear dye penetration in millimeters was the prespecified primary outcome. Because its distribution was non-normal and contained zero values, inference followed the balanced 3 × 2 factorial design using a two-factor permutation analysis based on the Freedman–Lane procedure with 100,000 permutations. The model included an additional conditioning method, adhesive system, and their interaction. F statistics are reported with permutation-derived *p* values, and partial η^2^ values describe effect magnitude. A six-group Kruskal–Wallis test was retained as a secondary omnibus sensitivity analysis. Descriptive values are reported as medians, interquartile ranges, and individual observations. The derived leakage grade was a supportive, exploratory secondary representation of the same measurement rather than an independent endpoint and was analyzed using a cumulative logit proportional-odds model. Interaction was tested by a likelihood-ratio comparison of models with and without the interaction terms; after the interaction was found non-significant, overall method and adhesive effects were evaluated in the additive model. Odds ratios with 95% confidence intervals are reported for model contrasts. The pairwise model-based contrasts and their confidence intervals were not adjusted for multiple comparisons and were interpreted as exploratory. Odds ratios were parameterized for higher leakage grades; for the first-listed condition versus the reference, an odds ratio below 1 indicates lower odds of being in a higher leakage grade. The previous Pearson chi-square subgroup analyses and numerical-grade ANOVA were not retained because of sparse cells, non-independent multiple testing, and the ordinal scale. Wilson 95% confidence intervals were calculated for the observed proportion of grade 0 specimens in each group. The significance level was α = 0.05.

## 3. Results

### 3.1. Distribution of Microleakage Grades

Microleakage was assessed in 90 specimens, with 15 specimens in each group. The grade counts were verified directly against the original specimen-level measurements before the ordinal analysis and are presented in Table 4. Overall, 34 specimens had grade 0, 50 had grade 1, and 6 had grade 2 microleakage; grades 3 and 4 were not observed. The Wilson 95% confidence intervals for the proportion with grade 0 were broad: Group 1, 26.7% (10.9–52.0%); Group 2, 20.0% (7.0–45.2%); Groups 3–5, 46.7% each (24.8–69.9%); and Group 6, 40.0% (19.8–64.3%). These intervals demonstrate substantial uncertainty around the observed proportions.

### 3.2. Primary Factorial Analysis of Linear Microleakage

The two-factor permutation analysis detected no statistically significant main effect of the additional conditioning method (F = 1.99, *p* = 0.142, partial η^2^ = 0.045), adhesive system (F = 0.28, *p* = 0.606, partial η^2^ = 0.003), or their interaction (F = 1.09, *p* = 0.341, partial η^2^ = 0.025) on maximum linear dye penetration (Table 5).

### 3.3. Descriptive Linear Microleakage and Sensitivity Analysis

Group medians ranged from 0.02 to 0.12 mm, with substantial overlap of the interquartile ranges and individual observations (Table 6 and Figure 2). The secondary six-group Kruskal–Wallis test also detected no statistically significant difference, with H(5, N = 90) = 5.75, *p* = 0.331.

### 3.4. Secondary Ordinal Analysis

Because grades 3 and 4 were absent and only six specimens had grade 2, the ordinal analysis had limited precision. The method × adhesive interaction was not statistically significant in the proportional-odds model (likelihood-ratio *p* = 0.754). In the additive model, the overall effect of additional conditioning method was not statistically significant (likelihood-ratio *p* = 0.053), whereas the adhesive-system effect was not significant (*p* = 0.763). Model-based odds ratios and 95% confidence intervals are shown in Table 7. These pairwise contrasts were unadjusted for multiplicity and are exploratory. The confidence interval excluding 1.0 for the Al_2_O_3_-versus-bur contrast and the non-significant omnibus method test are not contradictory because the single contrast and the two-degree-of-freedom factor test evaluate different hypotheses. The intervals were wide, consistent with the sparse upper category and limited ability to detect small effects.

## 4. Discussion

The present in vitro study evaluated maximum linear dye penetration at the aged composite–new composite repair interface after three additional conditioning procedures and the use of two adhesive systems. All specimens first underwent standardized removal with a diamond bur; therefore, the compared procedures represented additional bur roughening, additional Al_2_O_3_ airborne-particle abrasion, or additional Er:YAG irradiation, rather than three entirely independent preparation methods. The primary two-factor permutation analysis detected no statistically significant effect of additional conditioning method, adhesive system, or their interaction. The supportive, exploratory ordinal model likewise showed no significant interaction or adhesive effect, and the overall method effect was not statistically significant (*p* = 0.053). Composite repair nevertheless remains consistent with minimally invasive dentistry because it preserves sound tooth structure, reduces the risk of pulpal complications, and may slow the restorative cycle [5,6]. Loomans and Özcan similarly described intraoral repair as a conservative procedure that may prolong the service life of defective restorations when durable adhesion is achieved [7]. Bonding to aged composite remains challenging because water sorption, solubility, and hydrolytic degradation can alter the resin matrix and reduce the availability of reactive methacrylate groups [1,2,3,4]. Reviews have also emphasized the influence of composite age and mechanical pretreatment on repair bonding [8,9]. The present study extends this evidence by addressing interfacial sealing rather than mechanical strength alone.

The descriptive and model-based findings for Al_2_O_3_ abrasion require careful interpretation. Additional Al_2_O_3_ conditioning produced a numerically lower leakage distribution than additional bur roughening, and the model-based, unadjusted ordinal contrast for Al_2_O_3_ versus bur treatment indicated lower odds of a higher leakage grade. Its 95% confidence interval excluded 1.0, whereas the two-degree-of-freedom omnibus method test was not statistically significant and the primary continuous-outcome test was also not statistically significant. Because the contrast and omnibus tests evaluate different hypotheses, and because no multiplicity adjustment was applied, this single contrast is exploratory and requires confirmation rather than establishing superiority. Celik et al. reported lower leakage grades after 50-μm Al_2_O_3_ abrasion than after bur finishing [17]. Rashidi et al. found that both air abrasion and Er:YAG irradiation produced higher repair bond strength than bur preparation [32], and Bulut et al. observed the highest shear bond strength after airborne-particle abrasion [41]. Other studies have shown that responses to air abrasion, bur roughening, and laser treatment vary with the repair protocol, composite type, and use of silane [18,19,20,26]. These reports support the microretentive rationale for Al_2_O_3_ abrasion, but bond strength and interfacial sealing are related yet distinct outcomes. Mechanical tests quantify resistance to separation, whereas the present study quantified dye penetration along a repaired interface.

The Er:YAG groups also showed outcome- and adhesive-dependent numerical patterns, but individual linear measurements overlapped extensively with those in the other groups and no statistically significant Er:YAG effect was demonstrated. The available literature is similarly heterogeneous. Celiksoz et al. reported improved repair bond strength after Er:YAG irradiation [31] and later showed that different energy levels produced different outcomes [42]. In contrast, Barcellos et al. observed lower repair strength after Er:YAG treatment than after bur roughening or aluminum oxide abrasion [30]. Bektas et al. and Elsahn et al. further showed that aging, pulse energy, and repair-composite viscosity can modify laser-assisted repair performance [33,34]. Evidence from adhesive, cellular, and other dental-surface models also indicates that laser effects depend on wavelength, dose, material, and exposure conditions [35,36,37,38]. Pulse energy, frequency, pulse duration, water cooling, working distance, spot size, focal position, scanning movement, and composite composition may therefore contribute to divergent findings.

No statistically significant effect of the adhesive system was detected under the investigated conditions, in agreement with the microleakage findings of Cavalcanti et al. [12]. The role of universal and silane-containing adhesives in composite repair nevertheless remains debated. Altinci et al. reported reliable composite repair with a universal adhesive and possible benefits from airborne-particle abrasion or silane [21]; Chuenweravanich et al. found favorable durability after combining bur roughening, silane, and a universal adhesive [22]; and Gajski et al. observed no superiority of a silane-containing adhesive over a silane-free product [23]. Further investigations indicate that separate silanization, universal-adhesive formulation, mechanical pretreatment, and aging may affect bond strength and fatigue resistance [24,25,26,27], while other adhesive models demonstrate effects of application technique and substrate pretreatment [28,29]. Irmak et al., Celik et al., and Bandaru et al. likewise reported protocol-dependent mechanical outcomes [17,43,44]. Importantly, the present comparison involved two specific commercial products that differed in monomers, solvents, filler content, application steps, and the presence of incorporated silane. It did not isolate the chemical contribution of silane. The findings support the conclusion that a statistically significant difference between Clearfil SE Bond and Clearfil Universal Bond Quick 2 was not detected in this microleakage model.

Interpretation must also account for the characteristics of the leakage assessment. Dye penetration is widely used in laboratory studies [39], but the measured extent can depend on the tracer, immersion time, sectioning plane, magnification, and scoring method [14,15,16]. In the present study, the maximum linear extent of penetration was the primary continuous outcome, while an ordinal grade was used as a secondary representation. The numerical pattern was not identical for every group across these two summaries, illustrating how categorization can reduce information and alter apparent ranking when values are concentrated near the lower end of the scale. For this reason, the continuous and ordinal results should be viewed as complementary analyses rather than as independent endpoints. The restricted ordinal distribution further limited discrimination between protocols: 84 of 90 specimens had grades 0 or 1, only six had grade 2, and none had grades 3 or 4. This floor effect, together with the sample size, resulted in wide confidence intervals for grade 0 proportions and model contrasts. The absence of statistically significant differences should therefore not be interpreted as proof of genuine similarity. The two 1000-cycle procedures provided a standardized, moderate thermal challenge before and after repair, but they did not correspond to a defined duration of clinical service and did not reproduce long-term hydrolytic degradation. Thermocycling itself only approximates intraoral aging and may not produce homogeneous specimen temperatures [44]. The absence of mechanical loading may also have contributed to the generally low penetration values. Repeated loading could propagate interfacial defects that remain undetected after thermal challenge alone and might reveal different behavior among conditioning or adhesive protocols.

Several additional limitations should be acknowledged. The in vitro design did not reproduce saliva, biofilm, pH fluctuations, enzymatic degradation, occlusal loading, or the combined thermal and mechanical stresses of clinical function. Because every repair surface was first created with a diamond bur, the study cannot determine the effects of airborne-particle abrasion or Er:YAG irradiation used without prior bur preparation. Actual laser output and LED irradiance were not independently calibrated; nominal settings therefore may not exactly represent the energy delivered to each specimen. Surface morphology and roughness were not measured, so proposed micromechanical explanations cannot be directly linked to the observed leakage. Only one nanohybrid composite and two adhesive systems were evaluated, which limits generalization to other materials. Tooth type was not incorporated as a covariate; although the endpoint was confined to the standardized composite–composite interface, residual specimen-level variation cannot be excluded. In addition, the a priori calculation targeted a large six-group omnibus effect and was not specifically powered for smaller factorial interaction effects.

Future studies should verify laser and curing-light output at the specimen surface, control or record scanning speed, characterize surface topography, and combine microleakage assessment with repair bond-strength testing. Serial sectioning or three-dimensional imaging would better represent the spatial distribution of leakage, and repeated blinded measurements would allow examiner reliability to be quantified. Longer thermal aging combined with mechanical loading, larger samples planned for interaction effects, and evaluation of several composite and adhesive systems would improve precision and external validity. Within the present experimental conditions, the primary analysis did not detect statistically significant differences in interfacial sealing among the tested combinations. The exploratory, unadjusted ordinal contrast for additional Al_2_O_3_ conditioning may warrant further study, but the non-significant omnibus method test, uncertainty of the estimates, and non-significant primary analysis preclude a claim of superiority. Likewise, the absence of detected differences for the investigated Er:YAG protocol and adhesives should not be interpreted as evidence of equivalence or as an isolated test of silane efficacy.

## 5. Conclusions

Within the limitations of this in vitro study, no statistically significant effects of additional surface-conditioning method, adhesive system, or their interaction were detected for the primary outcome of maximum linear dye penetration. The supportive, exploratory secondary ordinal analysis also did not detect a statistically significant interaction or adhesive-system effect, and the overall method effect was not statistically significant (*p* = 0.053). These findings neither establish equivalence among the procedures nor isolate an effect of silane. They apply specifically to the tested materials, standardized bur-created repair surfaces, and experimental aging protocol.

## Figures and Tables

**Figure 1:**
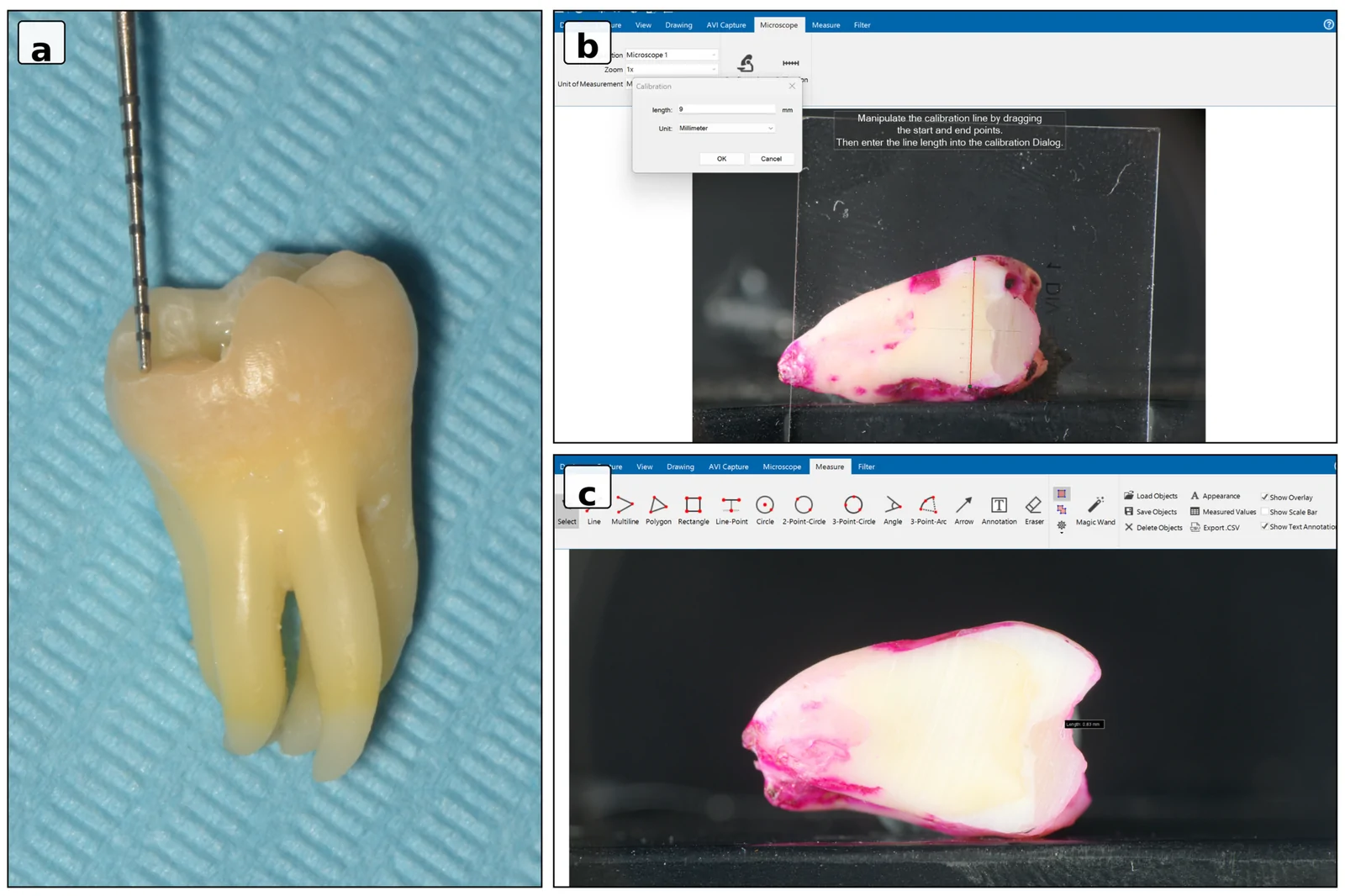
Experimental workflow for specimen preparation and microleakage assessment: (**a**) standardized Class II cavity preparation and verification of cavity dimensions using a periodontal probe; (**b**) calibration of the image-analysis software using a millimeter reference scale; (**c**) linear measurement of dye penetration along the aged composite–new composite repair interface.

**Figure 2:**
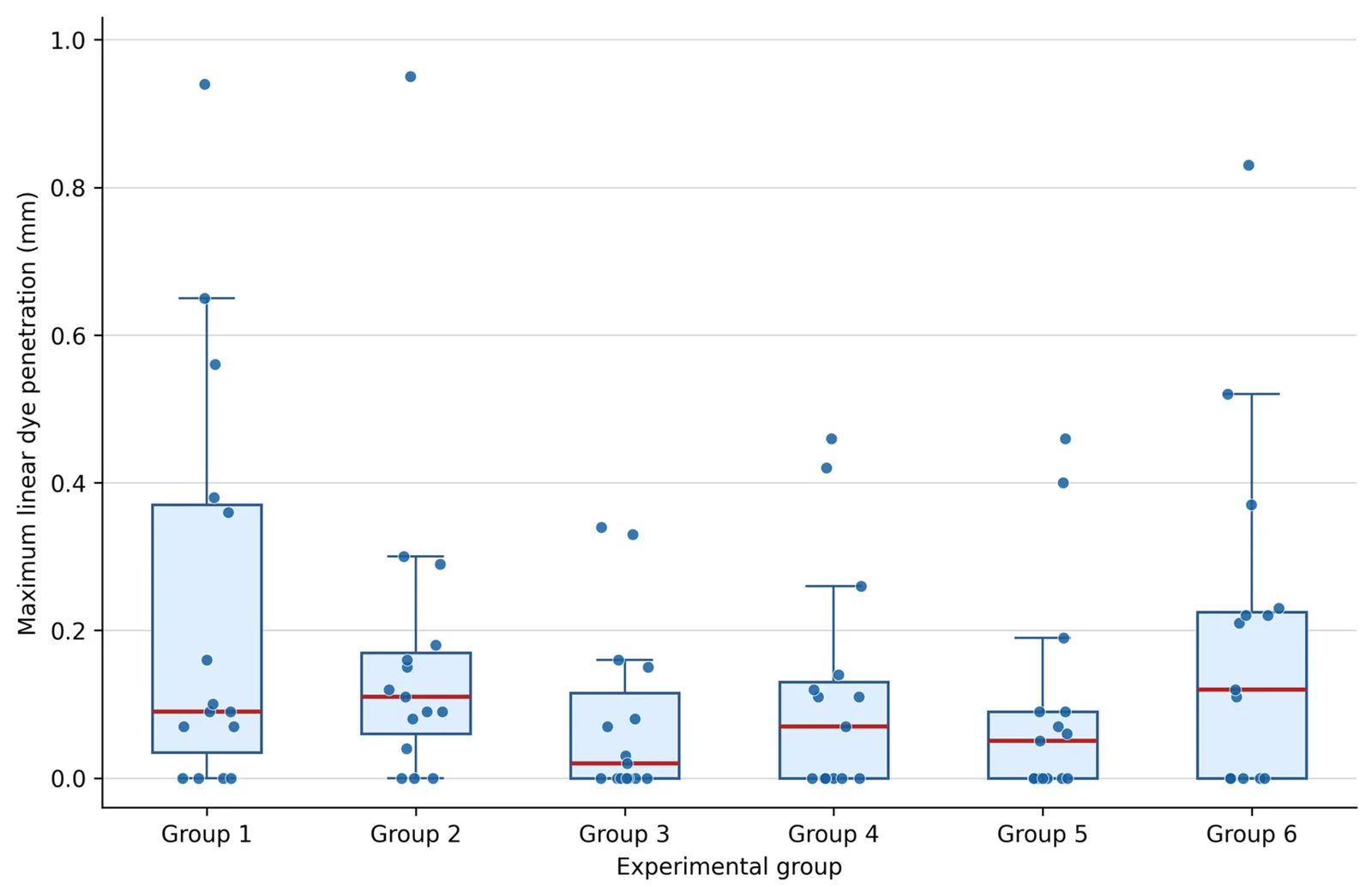
Distribution of maximum linear dye penetration in the six experimental groups. Individual observations are superimposed on box plots showing the median and interquartile range.

**Table 1:** Materials used in the study.

Material	Category	Chemical Composition	Manufacturer
CLEARFIL MAJESTY ES-2 Universal	Light-cured nanohybrid resin composite	Bis-GMA; hydrophobic aromatic and aliphatic dimethacrylates; silanated barium glass; prepolymerized filler; silanated colloidal silica; camphorquinone. Approx. 78 wt% filler, including 40 vol% inorganic filler; particle size 0.37–1.5 µm.	Kuraray Noritake Dental Inc., Tokyo, Japan
Clearfil SE Bond	Two-step self-etch adhesive	Primer: MDP, HEMA, hydrophilic dimethacrylate, water, photoinitiator/accelerator. Bond: MDP, Bis-GMA, HEMA, hydrophobic dimethacrylate, colloidal silica, photoinitiator/accelerator.	Kuraray Noritake Dental Inc., Tokyo, Japan
Clearfil Universal Bond Quick 2	One-step universal, silane-containing adhesive	Bis-GMA, hydrophilic amide monomers, ethanol, water, HEMA, MDP, colloidal silica, urethane tetramethacrylate, camphorquinone, accelerator, silane coupling agent, BAPO, sodium fluoride.	Kuraray Noritake Dental Inc., Tokyo, Japan

**Table 2:** Experimental groups.

Group	Additional Surface-Conditioning Method	Adhesive System	NUMBER of Specimens
1	diamond-bur roughening	Clearfil SE Bond	15
2	diamond-bur roughening	Clearfil Universal Bond Quick 2	15
3	50-µm Al_2_O_3_ airborne-particle abrasion	Clearfil SE Bond	15
4	50-µm Al_2_O_3_ airborne-particle abrasion	Clearfil Universal Bond Quick 2	15
5	Er:YAG laser irradiation	Clearfil SE Bond	15
6	Er:YAG laser irradiation	Clearfil Universal Bond Quick 2	15

**Table 3:** Ordinal microleakage grading scale.

Grade	Maximum Linear Dye Penetration
0	0.0 mm
1	>0.0 to ≤0.5 mm
2	>0.5 to ≤1.0 mm
3	>1.0 to <2.0 mm
4	≥2.0 mm

**Table 4:** Distribution of microleakage grades in all experimental groups.

Microleakage Grade	Group 1	Group 2	Group 3	Group 4	Group 5	Group 6	Total
grade 0	4	3	7	7	7	6	34
grade 1	8	11	8	8	8	7	50
grade 2	3	1	0	0	0	2	6
grade 3	0	0	0	0	0	0	0
grade 4	0	0	0	0	0	0	0
Total	15	15	15	15	15	15	90

**Table 5:** Two-factor permutation analysis of maximum linear dye penetration.

Effect	df	F	Permutation p	Partial η^2^
Additional conditioning method	2	1.99	0.142	0.045
Adhesive system	1	0.28	0.606	0.003
Method × adhesive system	2	1.09	0.341	0.025

**Table 6:** Median and interquartile range of maximum linear dye penetration (mm).

Statistic	Group 1	Group 2	Group 3	Group 4	Group 5	Group 6
Median	0.09	0.11	0.02	0.07	0.05	0.12
Q1	0.00	0.04	0.00	0.00	0.00	0.00
Q3	0.38	0.18	0.15	0.14	0.09	0.23

**Table 7:** Proportional-odds model for the derived ordinal microleakage grade.

Model Contrast	Odds Ratio	95% Confidence Interval
Additional Al_2_O_3_ abrasion vs. additional bur roughening	0.30	0.10–0.85
Additional Er:YAG irradiation vs. additional bur roughening	0.39	0.13–1.11
Clearfil Universal Bond Quick 2 vs. Clearfil SE Bond	1.14	0.50–2.60

Note: Odds ratios are oriented toward higher leakage grades; an odds ratio below 1 indicates lower odds of a higher grade for the first-listed condition relative to the reference. The model-based 95% confidence intervals are unadjusted, no multiplicity correction was applied, and these contrasts are exploratory.

## Data Availability

The original contributions presented in this study are included in the article. Further inquiries can be directed to the corresponding author.

## References

[1] Ferracane J.L. (2006). Hygroscopic and hydrolytic effects in dental polymer networks. Dent. Mater..

[2] Abdullah H.A., Jellil E.I., Jasim A.A., Baker E.Y. (2026). Comparative evaluation of sorption and solubility of contemporary light-cured resin cement, resin-modified glass ionomer, and self-adhesive resin luting cement in distilled water and artificial saliva. J. Stomatol..

[3] Dobrzyński W., Piszko P.J., Kiryk J., Kiryk S., Michalak M., Kotela A., Kensy J., Świenc W., Grychowska-Gąsior N., Matys J. (2025). Dental resin composites modified with chitosan: A systematic review. Mar. Drugs..

[4] Tyagi S., Jha P., Nikhil V. (2026). Effect of increasing tip distances of polywave light curing unit on microhardness profiles of bulk-fill composites containing different photoinitiator systems. J. Stomatol..

[5] FDI World Dental Federation (2017). FDI policy statement on Minimal Intervention Dentistry for managing dental caries. Int. Dent. J..

[6] FDIWorld Dental Federation (2020). Repair of restorations: Adopted by the General Assembly September, 2019, San Francisco, United States of America. Int. Dent. J..

[7] Loomans B.A.C., Özcan M. (2016). Intraoral repair of direct and indirect restorations: Procedures and guidelines. Oper. Dent..

[8] Neto H.N.M., Leite J.V.C., Medeiros J.M., Campos D.S., Ferreira Muniz I.d.A., De Andrade A.K.M., Duarte R.M., De Souza G.M., Lima R.B.W. (2024). Effect of surface treatments on bond strength of resin composite repair: A scoping review. J. Dent..

[9] Dieckmann P., Baur A., Dalvai V., Wiedemeier D.B., Attin T., Tauböck T.T. (2020). Effect of composite age on the repair bond strength after different mechanical surface pretreatments. J. Adhes. Dent..

[10] Galal Borhamy A., Al-Taee R., Osama Hisham H., Basha I., Nawaya S. (2026). Influence of adhesive strategy and dentin condition on shear bond strength: An in vitro study of sound and artificial caries-affected dentin. J. Stomatol..

[11] Basha E., Haddad M. (2025). Evaluation of bond strength of cinnamon essential oil-modified composite resin to dentin: An in vitro study. J. Stomatol..

[12] Cavalcanti A.N., Lavigne C., Fontes C.M., Mathias P. (2004). Microleakage at the composite-repair interface: Effect of different adhesive systems. J. Appl. Oral Sci..

[13] Shafiei F., Berahman N., Niazi E. (2016). Effect of finishing time on microleakage at the composite-repair interface. Open Dent. J..

[14] Mijkovic M., Dacic S., Popovic J., Nikolic M., Stosic N. (2026). Review of dye methods for microleakage assessment in restorative dentistry and endodontics. J. Esthet. Restor. Dent..

[15] Gupta K., Tandale A.B., Shetty S., Nihalani H. (2025). Confocal laser scanning microscopic analysis of microleakage in class II restorations: Zirconia-incorporated glass ionomer versus bulk-fill composite. J. Stomatol..

[16] Bahari M., Ajami A.A., Ebrahimi Chaharom M.E., Kahnamouei M.A., Katebi K., Aghazadeh A. (2024). Pre-heating decreases micro-leakage of bulk fill composite resins in dentin margins of class II cavities: An in vitro study. J. Stomatol..

[17] Celik C., Cehreli S.B., Arhun N. (2015). Resin composite repair: Quantitative microleakage evaluation of resin–resin and resin–tooth interfaces with different surface treatments. Eur. J. Dent..

[18] Elsaca N., Pardo-Díaz C., Atria P.J., Jorquera G., Turbino M.L., Sampaio C.S. (2023). Repair bond strength of aged composite resins using different surface treatment protocols. Int. J. Periodontics Restor. Dent..

[19] Ghavam M., Naeemi M., Hashemikamangar S.S., Ebrahimi H., Kharazifard M.J. (2018). Repair bond strength of composite: Effect of surface treatment and type of composite. J. Clin. Exp. Dent..

[20] Ahmadizenouz G., Esmaeili B., Taghvaei A., Jamali Z., Jafari T., Amiri Daneshvar F., Khafri S. (2016). Effect of different surface treatments on the shear bond strength of nanofilled composite repairs. J. Dent. Res. Dent. Clin. Dent. Prospects.

[21] Altinci P., Mutluay M., Tezvergil-Mutluay A. (2018). Repair bond strength of nanohybrid composite resins with a universal adhesive. Acta Biomater. Odontol. Scand..

[22] Chuenweravanich J., Kuphasuk W., Saikaew P., Sattabanasuk V. (2022). Bond durability of a repaired resin composite using a universal adhesive and different surface treatments. J. Adhes. Dent..

[23] Gajski P., Par M., Tarle Z., Marovic D. (2024). Effect of silane-containing adhesives on repair bond strength between fresh and aged composite materials: A pilot study. Materials.

[24] Çakir N.N., Demirbuga S., Balkaya H., Karadaş M. (2018). Bonding performance of universal adhesives on composite repairs, with or without silane application. J. Conserv. Dent..

[25] Stape T.H.S., Tulkki O., Salim I.A., Jamal K.N., Mutluay M.M., Tezvergil-Mutluay A. (2022). Composite repair: On the fatigue strength of universal adhesives. Dent. Mater..

[26] Hashim H., Abd-Alla M.H. (2022). Silanizing effectiveness on the bond strength of aged bulk-fill composite repaired after sandblasting or bur abrasion treatments: An in vitro study. Clin. Cosmet. Investig. Dent..

[27] Kanzow P., Baxter S., Rizk M., Wassmann T., Wiegand A. (2019). Effectiveness of a universal adhesive for repair bonding to composite and amalgam. J. Oral Sci..

[28] Eltobgy O., Abd-Elhady A., Abdelatty A., Elmanakhly A., Mohamed H., Basha I., Gabr E. (2025). The effect of ultrasonic agitation on enamel bond strength of universal adhesives with light-cure versus self-cure activation: An in vitro comparative study. J. Stomatol..

[29] Jain S., Srinidhi S.R. (2025). Comparative evaluation of the efficacy of bonding agents in cervical cavities after using desensitizing agents: An in vitro study. J. Stomatol..

[30] Barcellos D.C., Santos V.M.M., Niu L.N., Pashley D.H., Tay F.R., Pucci C.R. (2015). Repair of composites: Effect of laser and different surface treatments. Int. J. Adhes. Adhes..

[31] Celiksoz O., Yilmaz N.A., Balin E. (2022). Effect of Er:YAG laser on repair bond strength of a nano-hybrid composite. J. Stomatol..

[32] Rashidi M., Berangi S., Chiniforush N., Ahmadi E., Ranjbar Omrani L. (2022). Microtensile repair bond strength of a composite after accelerated artificial aging: Effect of air abrasion, bur, Er:YAG laser, two-step self-etch bonding, and universal bonding repair system. J. Lasers Med. Sci..

[33] Bektas Ö.Ö., Eren D., Herguner Siso S., Akin G.E. (2012). Effect of thermocycling on the bond strength of composite resin to bur and laser treated composite resin. Lasers Med. Sci..

[34] Elsahn N.A., Alshouli M.A., Bahdar S.A., Zafar M.S. (2026). Effect of laser pulse energy and repair composite viscosity on the repair shear bond strength of aged bulk-fill resin composites. Front. Dent. Med..

[35] Florczak-Matyjek A., Nikodem A., Kensy J., Matys J., Grzech-Leśniak K. (2025). The efficacy of erbium-ion, diode, and CO_2_ lasers in debonding attachments used during overlay orthodontic treatment and the risk of hard tooth tissue damage compared to traditional methods—An in vitro study. Photonics.

[36] Świenc W., Kiryk J., Michalak M., Majchrzak Z., Laszczyńska M., Kiryk S., Grychowska-Gąsior N., Nawrot-Hadzik I., Matys J., Dobrzyński M. (2025). The effect of laser surface treatment on the bond strength of adhesive materials to primary teeth: A systematic review. Materials.

[37] Fiegler-Rudol J., Matys J., Wojtyczka R.D., Skaba D.R., Wiench R. (2026). Dose-dependent effects of Er:YAG laser on human fibroblast proliferation, cell death, and γH2AX DNA damage signaling. Ann. Acad. Med. Siles..

[38] Sobierajska P., Dobrzyński M., Grzech-Leśniak K., Sekula K., Szymanski D., Zakrzewski W., Dudek K.D., Matys J., Szymonowicz M., Wiglusz K. (2026). The effect of the Er^3+^:YAG laser decontamination process on the surface of titanium alloys, depending on the exposure parameters. Materials.

[39] Bilgrami A., Alam M.K., Qazi F.U.R., Maqsood A., Basha S., Ahmed N., Syed K.A., Mustafa M., Shrivastava D., Nagarajappa A.K. (2022). An in-vitro evaluation of microleakage in resin-based restorative materials at different time intervals. Polymers.

[40] Eliasson S.T., Dahl J.E. (2020). Effect of thermal cycling on temperature changes and bond strength in different test specimens. Biomater. Investig. Dent..

[41] Bulut A., Bala O., Akgül S. (2024). The effect of different treatment protocols on shear bond strength in resin composite restoration repair. BMC Oral. Health.

[42] Celiksoz O., Recen D., Peskersoy C. (2023). The effect of different energy levels of the Er:YAG laser on the repair bond strength of a nanohybrid composite resin. Proc. Inst. Mech. Eng. H J. Eng. Med..

[43] Irmak O., Celiksoz O., Yilmaz B., Yaman B.C. (2017). Adhesive system affects repair bond strength of resin composite. J. Istanb. Univ. Fac. Dent..

[44] Bandaru P., Bolla N., Garlapati R., Vemuri S., Bandaru Y. (2021). Repair bond strength of aged resin composite using different surface treatments and bonding protocols. Int. J. Dent. Mater..

